# Direct patient-physician communication via a hepatitis C hotline facilitates treatment initiation in patients with poor adherence

**DOI:** 10.1007/s00508-020-01790-y

**Published:** 2020-12-22

**Authors:** Lisa Steininger, David Chromy, David Bauer, Benedikt Simbrunner, Teresa Binter, Philipp Schwabl, Caroline Schmidbauer, Michael Trauner, Michael Gschwantler, Mattias Mandorfer, Thomas Reiberger

**Affiliations:** 1 Division of Gastroenterology and Hepatology, Department of Internal Medicine III, Medical University of Vienna, Waehringer Guertel 18–20, 1090 Vienna, Austria; 2 Vienna HIV & Liver Study Group, Vienna, Austria; 3 Division of Immunology, Allergy and Infectious Diseases, Department of Dermatology, Medical University of Vienna, Vienna, Austria; 4 Department of Internal Medicine IV, Wilhelminenspital, Wiener Krankenanstaltenverbund (KAV) der Stadt Wien, Vienna, Austria

**Keywords:** Tele-medicine, Elimination, Compliance, Direct acting antivirals, People who inject drugs

## Abstract

**Background:**

Despite the availability of effective and well-tolerated direct acting antivirals (DAAs) against hepatitis C virus (HCV) infection, a substantial number of HCV patients remain untreated. Novel strategies targeting HCV patients with poor adherence are urgently needed to enable HCV elimination.

**Methods:**

We implemented a physician-operated HCV hotline (HCV-Phone) that was promoted within the patient community and referral networks. Previously diagnosed HCV patients were contacted via the HCV-Phone and offered low-barrier access to DAA therapy. Patients/referring physicians could directly call or send messages to the HCV-Phone. The HCV-Phone related and unrelated visits as well as DAA treatment initiations throughout 2019 were documented. Patients were followed until October 2020. This study analyzed treatment initiation, adherence to scheduled visits and outcomes in patients in whom management was assisted by the HCV-Phone.

**Results:**

Out of 98 patient contacts via the HCV-Phone 74 attended treatment assessment at our clinic. While 15 (20%) patients were HCV-RNA negative and 1 (1%) patient did not initiate therapy, 58 patients were recruited for DAA therapy via the HCV-Phone. A total of 21 additional patients who started DAAs without HCV-Phone assistance required the use of the HCV-Phone infrastructure later on during treatment, resulting in a total of 79 HCV-Phone related DAA therapies. The poor adherence of patients previously diagnosed with HCV at our clinic is underlined by the long duration from HCV diagnosis to DAA therapy of median 37.0 months (IQR 2.7–181.1 months). A total of 55 (70%) HCV patients achieved a sustained virological response (SVR), 5 (6%) discontinued therapy, 1 (1%) had a reinfection, while 10 (13%) and 8 (10%) patients were lost during DAA therapy or follow-up, respectively.

**Conclusion:**

The implementation of a physician-operated phone hotline for patients with HCV infection facilitated treatment initiation in an HCV population with poor adherence. Mainly due to losses to follow-up, the SVR rate remained suboptimal with 70%.

## Introduction

With an estimated global prevalence of 71.1 million people affected, chronic hepatitis C virus (HCV) infection remains one of the leading causes of cirrhosis, liver-related death and hepatocellular carcinoma worldwide [1]. In Europe and the USA the vast majority of HCV infections are observed in people who inject drugs (PWID) and HIV positive men who have sex with men (MSM) engaging in high-risk sexual practices [2, 3].

In 2016, the World Health Organization (WHO) defined targets for reducing the disease burden, with the goal of eliminating HCV until 2030 [4]. However, this requires improved screening strategies for HCV infection, facilitated access to medication by better linkage to care as well as patient education for prevention of reinfections [5]. Interferon-free direct acting antivirals (DAA) achieve sustained virologic response (SVR) rates >95%, both in patients with HCV monoinfection and HCV-HIV co-infection [6–8], including the subgroup of patients with advanced fibrosis [9]. In contrast to previous interferon (IFN)-based regimens [10], IFN-free DAA-based treatments are well-tolerated and do not compromise health-related quality of life [11]. Although the initiation of treatment in PWID has reportedly increased since the introduction of DAA-based therapies, especially people with ongoing drug abuse still show low rates of treatment initiation and suboptimal compliance [12]. Among Viennese HIV positive PWID, the prevalence of HCV infections remained stable over the last years, while it increased in HIV positive MSM [13] due to high-risk sex practices [14]. Similar observations were made in other European countries, for example, in the Netherlands, where a rising incidence of HCV infections in HIV negative MSM using pre-exposure prophylaxis (PrEP) has been observed [15]. Hence, it is of great importance to provide low barrier access to HCV counselling for patients at high risk of viral infection and transmission in order to improve early screening for HCV infection and facilitate access to DAA therapy.

Increasing access to the internet and smartphones has created a multitude of novel options to improve communication between hard to reach patient populations and medical care providers. High rates of access to mobile phones and the internet have been described in patients with substance abuse [16, 17].

Telemedicine-based HCV treatment has previously been reported to increase the initiation of DAA treatments and result in high SVR rates [18]. Promoting information through social media has been shown to increase awareness and willingness for testing of HCV or HIV infection and improved linkage to care in specific risk groups [19, 20]. We hypothesized that HCV patients would benefit from treatment strategies involving mobile phones as they enable easy and direct access to HCV counselling and enable patients to schedule appointments and/or receive reminders for prescription renewal, treatment intake or for follow-up appointments.

Thus, we have established a direct patient-physician HCV-Phone hotline at our HCV treatment center in January 2019. The aim of our study was to analyze treatment initiation, adherence to scheduled visits and outcomes in HCV-infected patients at our treatment center in 2019, with a focus on HCV patients in whom management was assisted by a direct patient-physician telephone line (i.e. the HCV-Phone).

## Patients and methods

### Study design and recruitment strategy

All HCV-RNA positive patients attending the HCV clinic at the Medical University of Vienna in 2019 were included in this study. Those HCV patients previously diagnosed and managed at our clinic who still showed viremia at their last HCV-RNA PCR test before 2019 or those for whom SVR12 was not previously documented, were identified from medical records and invited for a treatment evaluation visit.

### Establishment of a direct patient-physician telephone line

In January 2019 we launched a HCV hotline (HCV-Phone), specifically targeted at individuals with HCV infection that was operated by a physician at our treatment center during regular working hours. It was promoted through a homepage (www.hep-c-hotline.at), flyers and our network of referral centers/physicians. Via this HCV hotline, referring physicians as well as patients were able to schedule appointments at our clinic or receive advice regarding HCV infections. Most importantly, access to clinical visits and subsequent initiation of HCV treatment was enabled without any additional barriers arising through administrative/bureaucratic issues.

In order to improve adherence and compliance to treatment, text messages reminding patients of their appointments were sent out daily to all patients with an appointment that day. Importantly, the HCV-Phone also had a mailbox that provided patients and/or referring centers/physicians with the opportunity to leave a message and/or number. The physician operating the HCV-Phone answered the messages and called back the numbers of missed calls on the next possible occasion.

The HCV-RNA positive patients, as described above, were actively called and invited to treatment evaluation visits via the HCV-Phone. Importantly, in cases of no answer by the respective patient a message with the offer for a call back option was placed at the respective patient’s mailbox.

### Treatment evaluation visits

All visits of HCV patients at our specialized HCV clinic between January 2019 and December 2019 were recorded. It was recorded how many HCV patients were seen after (i) they were actively called and invited to the clinic or (ii) if they were scheduled for an appointment after self-initiated contact or by a referral center/physician or (iii) if the appointment scheduled was not related to any previous contact via the HCV-Phone. At the treatment evaluation visit the patients underwent a confirmation HCV-RNA PCR and HCV genotype (GT) testing together with liver stiffness measurement [21].

### HCV treatment and study endpoints

The DAA treatment was selected based on local reimbursement policies and current treatment recommendations [22]. Routine check-up visits were scheduled every 4 weeks during treatment, at the end of treatment and after the end of treatment, in order to evaluate sustained virological response after 4 weeks and 12 weeks (i.e., SVR4 and SVR12, respectively). Patients were followed until October 2020 in order to record the outcomes of treatments initiated in 2019. Patients who did not attend our clinic at week 4 after the end of treatment but achieved SVR12 were also considered to have achieved SVR4. Treatment inititation and the rate of SVR4 comprised the primary efficacy endpoints.

### Patient parameters

Data regarding patient characteristics were collected from medical records. The HCV GT was determined with VERSANT® HCV Genotype 2.0 Assay Line Probe Assay (LiPA, Siemens Healthcare Diagnostics, Tarrytown, NY, USA) and HCV-RNA was quantified with Abbott RealTime HCV assay (Abbott Molecular, Des Plaines, IL, USA), which is capable of detecting and quantifying HCV RNA to a lower limit of 12 IU/mL.

### Liver stiffness measurement

Liver stiffness was measured with a Fibroscan® (Echosens, Paris, France), and either the M‑probe or XL-probe were used. Advanced fibrosis (F3) and cirrhosis (F4) were defined by liver stiffness values of 9.5–12.4 kPa and of ≥12.5 kPa, respectively.

### Statistical analysis

We used SPSS statistics version 26 (SPSS, IBM, Armonk, NY, USA) for statistical analyses. Continuous variables are presented as mean (SD) or median (IQR), while nominal parameters are shown as counts or proportions of patients. For the comparison of parametrically distributed continuous variables, we used the independent sample t‑test and for non-parametric variables, the Mann-Whitney U-test.

Depending on sample size, nominal variables were compared with either the χ^2^-test or Fisher’s exact test. For all tests, statistical significance was determined by a two-sided *p*-value of <0.05.

### Ethics

The Declaration of Helsinki was acknowledged throughout all aspects of this study. The local ethics committee of the Medical University of Vienna approved the study (EC number: 1968/2018).

## Results

### Patient population (Fig. 1; Table 1)

Our medical records included 167 subjects, who were still HCV-RNA viremic at their last clinical contact or who did not have documented SVR12 before 2019. Among those, 60 patients (36%) were successfully contacted via the HCV-Phone and invited for a treatment evaluation visit. Of these 60 patients 42 (72%) were HIV coinfected, 11/60 (18%) HCV infected patients were treatment-experienced and 7/60 (12%) had documented HCV antibodies but were treatment-naive. Additionally, 38 patients were referred to our treatment center via the HCV-Phone by external care providers or called the hotline themselves. In total, 98 patients were invited for treatment evaluation visits via the HCV-Phone; however, only 74 patients attended their scheduled HCV-Phone-related treatment evaluation visits during the study period. In addition, 21 patients who did not have HCV-Phone-related treatment evaluation used the HCV-Phone to arrange drug prescriptions, send reminders for treatment intake or recall for follow-up appointments during HCV treatment.

**Fig. 1:**
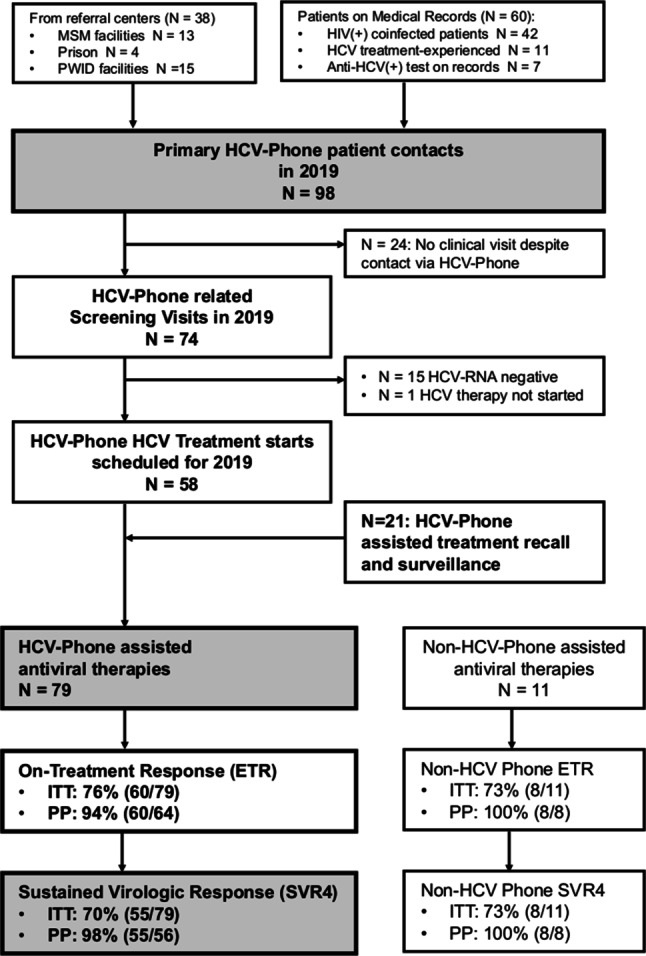
Patient flow chart. Out of 60 patients who were invited to a treatment evaluation visit by the HCV-Phone and 38 patients who were referred from other physicians or called the HCV-Phone themselves, 74 patients had HCV-Phone related treatment evaluation visits at our clinic. Of these 74 patients 1 did not start treatment until October 2020. In total, 79 patients underwent DAA therapy supported by the HCV-Phone (including 21 patients who were not started via the HCV-Phone but supported during therapy with the help of the HCV-Phone). Ultimately, 55 patients achieved sustained virologic response 4 weeks after end of therapy (SVR4), corresponding to an intention-to-treat SVR4 rate of 70% and a per-protocol SVR4 rate of 98%. *MSM* men who have sex with men, *PWID* people who inject drugs, *HIV* human immunodeficiency virus, *HCV* hepatitis C virus, *ETR* end of treatment response, *ITT* intention-to-treat analysis, *PP* per-protocol analysis, *SVR* sustained virological response

**Table 1:** Characteristics of HCV patients treated with DAAs in 2019

	DAA therapy related to HCV-Phone, *n* = 79	DAA therapy unrelated to HCV-Phone, *n* = 11	*p*-value
*Sex, male/female (% male)*	59/20 (75%)	8/3 (73%)	1.0
*Age, years, mean (SD)*	41.9 (15.4)	44.6 (27.0)	0.671
*BMI, median kg/m*^2^ *(IQR)*	23.3 (21.0–26.3)	23.9 (20.8–25.6)	0.775
*Treatment naïve, n (%)*	58 (73%)	10 (91%)	0.282
*HCV reinfection, n (%)*	11 (15%)	0 (–)	0.347
*HIV/HCV coinfection, n (%)*	41 (52%)	5 (45%)	0.689
*Genotype, n* (%)			
1a 1b	42 (53%) 14 (18%)	4 (36%) 1 (9%)	0.296 0.683
2	3 (4%)	0 (–)	1.0
3	14 (18%)	6 (55%)	0.013
4	6 (8%)	0 (–)	1.0
*Route of transmission, n (%)*			
IDU	43 (54%)	5 (45%)	0.576
MSM	16 (20%)	0 (–)	0.202
Other/unknown	20 (25%)	6 (55%)	0.072
*Liver stiffness, median kPa (IQR)*	6.1 (5.1–8.7)	5.9 (5.3–11.6)	0.751
*Liver cirrhosis, n (%)*	10 (13%)	2 (18%)	0.637
*HCV-RNA, median IU/ml (IQR)*	1.25 × 10^6^ (1.69 × 10^5^–3.45 × 10^6^)	3.60 × 10^6^ (1.37 × 10^5^–1.24 × 10^7^)	0.175
*Bilirubin, median mg/dL (IQR)*	0.44 (0.30–0.61)	0.43 (0.30–0.56)	0.743
AST, median U/L (IQR)	39 (28–75)	40 (23–51)	0.156
ALT, median U/L (IQR)	41 (24–81)	42 (15–73)	0.477
GGT, median U/L (IQR)	61 (30–133)	71 (20–134)	0.705

*DAA* direct acting antiviral, *IQR* interquartile range, *BMI* body mass index, *HCV* hepatitis C virus, *IDU* intravenous drug abuse, *MSM* men who have sex with men, *AST* aspartate aminotransferase, *ALT* alanine aminotransferase, *GGT* gamma-glutamyltransferase

Out of 74 subjects who had an HCV-Phone associated screening visit 15 (20%) had undetectable levels of HCV-RNA when evaluated for treatment. Out of these 74 patients 54 (78%) were started with antiviral therapy by the help of the HCV-Phone and in total 79 patients underwent HCV treatment assisted by the HCV-Phone. Importantly, one of these patients was treated with IFN for 48 weeks (and not with DAA), due to a comorbidity that also required IFN therapy anyway and explicit patient preference but was also included into our study cohort. For simplified reading and due to the fact that only 1 out of 79 patients did not receive DAA, we still refer to it as HCV-Phone-assisted DAA therapy. Thus, 4 patients were not started with DAA in 2019 despite contacts via the HCV-Phone that aimed to link these patients to care and HCV-DAA therapy; however, 3 of these 4 patients (75%) patients had finally started of DAA therapy later in 2020 and the other patient was imprisoned during his treatment evaluation visit at our clinic and thus, DAA treatment could not be initiated for this patient.

Among the HCV patients who were previously diagnosed with HCV and contacted via the HCV-Phone, the median time (IQR) between the initial HCV diagnosis and treatment initiation was 37.0 months (2.7–181.1 months). The median duration (IQR) between the last contact at our clinic before 2019 and the treatment evaluation visit was 5.6 months (2.0–9.5 months).

Additionally, 19 patients with suspected HCV infections visited our outpatient clinic in 2019 and were not related to any contact via the HCV-Phone: 3/19 (16%) showed spontaneous clearance of the HCV infection, 16/19 (84%) were HCV viremic and required treatment, 4 out of 16 (25%) viremic patients did not attend any further visits after SCR and 1/16 (6%) viremic patients started treatment at another HCV treatment center. Finally, 11/16 (69%) HCV patients received DAA therapy unrelated to any HCV-Phone contact at our clinic in 2019.

The characteristics of the 79 patients who underwent HCV therapy supported by the HCV-Phone were compared to the 11 patients undergoing DAA therapy in 2019, not related to the HCV-Phone project (Table 1).

### Patient characteristics and HCV-DAA therapies (Fig. 1; Table 1)

The following 90 treatments were initiated: glecaprevir/pibrentasvir in *n* = 51 patients (57%), grazoprevir/elbasvir in *n* = 14 (16%) patients, and sofosbuvir/velpatasvir in *n* = 24 (27%) patients. Due to a comorbidity and explicit patient preference, *n* = 1 (1%) patient was treated with IFN for 48 weeks.

Most patients with DAA therapy related to the HCV-Phone were male (59/79; 75%), and the mean age (SD) was 41.9 years (15.4 years), 58 out of 79 (73%) patients were treatment-naïve and 21/79 (27%) were treatment-experienced. Importantly, 11/79 (15%) patients had confirmed reinfection with HCV. Among patients that initiated contact with the HCV-Phone, 39% (15/38) were PWID, 34% (13/38) were MSM, and 11% (4/38) were former prisoners. About half the patients in the HCV-Phone group were HIV-HCV coinfected (41/79, 52%). HCV genotype (GT) 1a was the most common with 53% (42/79) in the HCV-Phone group and the main route of HCV transmission was intravenous drug abuse (IDU, 43/79, 54%). The median liver stiffness (IQR) in the HCV-phone associated group was 6.1 kPa (5.1–8.7 kPa), and the prevalence of advanced fibrosis (>9.5 kPa) and of cirrhosis was 19% (15/79) and 13% (10/79), respectively.

While in the non-HCV-Phone associated group GT 3 was more common (6/11; 55%; *p* = 0.013) than in the HCV-Phone associated group, there was no other significant difference in patient characteristics between both groups.

### Treatment initiation and compliance with clinical visits (Table 2; Fig. 2a)

Only *n* = 1 (1/59; 2%) of HCV-RNA viremic patients who had a HCV-Phone associated treatment evaluation visit did not start DAA therapy until October 2020. Importantly, significantly more HCV-RNA viremic patients in the non-HCV-Phone group (31%, 5/16) did not start DAA treatment in 2019 (*p* < 0.001). Of these, 3/16 (19%) patients were in contact with the HCV-Phone later but did not attend any further visits, 1/16 (6%) planned on starting treatment at another facility and 1/16 (6%) did not respond to any telephone calls or messages.

**Table 2:** Outcomes of the initiated DAA therapies

	DAA therapy related to HCV-Phone, *n* = 79	DAA therapy unrelated to HCV-Phone, *n* = 11	*p*-value
*End of treatment, n (%)*			
Lost during therapy	10 (13%)	3 (27%)	0.194
Therapy discontinued	5 (6%)	0 (–)	1.0
Full therapy	64 (81%)	8 (73%)	0.687
ETR (% ITT)	60/79 (76%)	8/11 (73%)	1.0
ETR (% PP, i.e. full therapy)	60/64 (94%)	8/8 (100%)	1.0
*4 weeks after end of treatment, SVR4 visit, n (%)*			
Lost during follow-up^a^	8 (10%)	0 (–)	0.589
Relapse/reinfection^b^	1 (1%)	0 (–)	1.0
SVR4 (% ITT)	55/79 (70%)	8/11 (73%)	1.0
SVR4 (% PP)	55/56 (98%)	8/8 (100%)	1.0

*DAA* direct acting antivirals, *ETR* end of treatment response, *ITT* intention-to-treat analysis, *SVR* sustained virological response, *PP* per-protocol analysis

^a^After end of treatment

^b^HCV-RNA relapse/reinfection between end of treatment and SVR12

**Fig. 2:**
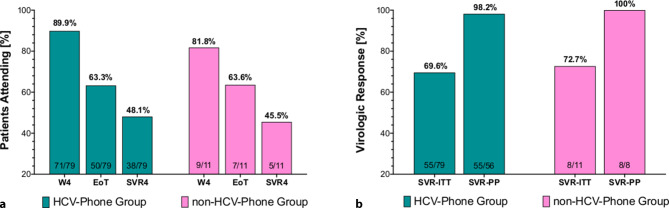
**a** Adherence to scheduled visits at weeks 3–4, at the end of treatment and 4 weeks post treatment (SVR4). Comparing both groups, 82% (9/11) of the non-HCV-Phone associated group vs. 87% (69/79) of the HCV-Phone associated group attended their scheduled visits at week 3–4 during treatment (*p* = 0.637), 64% (7/11) vs. 63% (50/79) attended the planned visits at the end of treatment (*p* = 1), and 45% (5/11) vs. 38% (38/79) had a visit at week 4 (SVR4) after treatment (*p* = 0.869). **b** Treatment outcomes. In the intention-to-treat analysis 55/79 (70%) patients of the HCV-Phone related group compared to 8/11 (73%) patients of the non-HCV-Phone related group achieved SVR4 (*p* = 1). When analyzed as per-protocol (PP), 55/56 (98%) and 8/8 (100%) achieved SVR4 (*p* = 1), respectively. *W* week, *EoT* end of treatment, *SVR* sustained virological response, *HCV* hepatitis C virus, *ITT* intention-to-treat analysis, *PP* per-protocol analysis

Comparing the 79 patients undergoing HCV-Phone supported DAA therapy to the 11 patients without HCV-Phone support, there was no statistically significant difference in adherence to scheduled clinical visits after initiation of treatment: 71/79 (90%) patients of the HCV-Phone-related group and 9/11 (82%) of the HCV-Phone-unrelated group attended their scheduled visit at the end of the first month of therapy (DAA treatment week 4; *p* = 0.352). A similar proportion of patients had a visit at the end of treatment with 50/79 (63%) in the HCV-Phone group and 7/11 (64%) in non-HCV-Phone group (*p* = 1). At week 4 after treatment/SVR4 both groups showed comparable adherence to planned visits (38/79, 48% vs. 5/11, 45%, *p* = 0.869).

### Treatment outcome per intention-to-treat and per-protocol (Table 2; Fig. 2b)

In total, 10/79 (13%) patients of the HCV-Phone group and 3/11 (27%; *p* = 0.194) of the HCV-Phone-unrelated group were lost during therapy, 5 out of 79 (6%) patients discontinued HCV-Phone-associated DAA therapy while 64/79 (81%) completed therapy. In comparison, 8/11 (73%) patients completed the HCV-Phone-unrelated DAA therapy (*p* = 1.0). The intention-to-treat (ITT) analysis showed that 60/79 (76%) and 8/11 (73%) of the HCV-Phone-related vs. HCV-Phone-unrelated patients achieved an end of treatment response (ETR), while 55/79 (70%) and 8/11 (73%), respectively, achieved SVR4.

When analyzed as per-protocol (PP), the ETR rates were 94% (60/64) in the HCV-Phone group and 100% (8/8) in the HCV-Phone-unrelated group (*p* = 1) and 98% (55/56), as well as 100% (8/8) achieved SVR4, respectively.

Eight out of 79 patients (10%) of the HCV-Phone group and 0/11 patients of the HCV-Phone-unrelated group were lost during follow-up after the end of treatment (*p* = 0.589), while 1/79 (1%) patients of the HCV-Phone-related group had a reinfection, with a different HCV-GT after achieving ETR but prior to the visit 12 weeks after cessation of therapy (i.e., the SVR12 visit).

## Discussion

In January 2019 a physician-operated hotline (HCV-Phone) targeting people with HCV infections, was launched at the HCV treatment center at the Vienna General Hospital. The main goals of this hotline were to enable direct communication between our HCV expert physicians and patients as well as referring physicians/networks, to facilitate access to HCV screening and to enable low barrier access to HCV treatment. A key feature of the HCV-Phone was the option to send and receive text and voice messages. Nonsimultaneous communication enabled us to stay in contact, schedule appointments, or remind patients of their clinical visits without the need for a telephone call, which in our experience is often tricky to schedule in a population of HCV patients with suboptimal adherence. Especially in patients with infrequent adherence to scheduled visits, as often observed in PWID [23], text messages were frequently used as a primary form of contact in our patient cohort.

A total of 60 HCV viremic patients from our medical records were successfully contacted by the HCV-Phone, and 38 patients were referred to our clinic via the HCV-Phone by cooperating physician networks.

An additional number of 21 patients underwent HCV treatment evaluation unrelated to the HCV-Phone but used the service of the HCV-Phone later on during DAA therapy. Finally, 79 patients (i.e., 88%, corresponding to the majority of patients who were receiving DAAs at our center in 2019) received HCV therapy supported by the HCV-Phone.

Treatment initiation was excellent in the HCV-Phone-related group with only 7% (4/59) HCV-RNA viremic patients not starting DAA treatment in 2019. Importantly, three of these four patients started DAA therapy early in 2020. By comparison, as many as 31% (5/16) HCV-RNA viremic patients in the non-HCV-Phone group did not start DAA therapy.

In total 90 DAA treatments were initiated at our treatment center until October 2020 after treatment evaluation in 2019, of which the majority (88%, 79/90) were HCV-Phone related. There were no significant differences in the demographic characteristics between the HCV-Phone and the non-HCV-Phone group. An IDU was the most common suspected route of transmission (54% and 45%, respectively; *p* = 0.576) in both groups.

Both patient groups achieved similar SVR4 rates with 70% (55/79) and 73% (8/11) in the HCV-Phone-related and HCV-Phone-unrelated group, respectively.

While treatment initiation in the HCV-Phone group was better, both groups showed similar adherence to scheduled treatment visits. When treatment outcomes were evaluated per-protocol, i.e. in patients undergoing HCV-RNA PCR testing at the scheduled visits, the SVR4 results were excellent with 98% (55/56) and 100% (8/8), respectively, confirming the high efficacy of these regimens. Only one patient in the HCV-Phone group was reinfected by another HCV-GT between the end of treatment and SVR12, most likely due to ongoing IDU. These results are in line with previous studies, where SVR rates >90% in patients with HCV monoinfection and HIV coinfection treated with DAA were reported [24–26].

Our HCV population that was contacted via the HCV-Phone and invited to a treatment evaluation visit seemed to have poor adherence and weak linkage to care earlier, as the median time to treatment after the initial HCV diagnosis was 37.0 months and the median time between the last contact before 2019 and the treatment evaluation visit was 5.6 months. We concluded that the use of a physician-operated HCV-Phone hotline facilitates linkage to care and improves adherence to enable a considerable proportion of these HCV patients access to DAA therapy; however, only 36% of patients (60/167) from our medical records who were either previously diagnosed or previously treated but without confirmed SVR12 could be contacted and invited to a treatment evaluation visit via the HCV-Phone. The other 107 patients (64%) could not be contacted due to missing or incorrect/changed contact data. As highlighted before, especially PWID and MSM should be assigned to a high priority patient population to be screened for HCV infection. Although treatment initiation in PWID is increasing since the implementation of IFN-free treatment regimens [12], our findings further underline that additional efforts are required to improve linkage to care, adherence to treatment and scheduled visits in this population of HCV patients. Intensified cooperation with other facilities, which might be attended more frequently by these patients (e.g. general practitioners or HIV treatment centers) or peer education through patients under HCV treatment could be useful to address this population with suboptimal adherence. Strategies, such as directly observed DAA therapy alongside opioid substitution therapy in HCV patients with poor compliance have already been demonstrated to be effective in increasing adherence to and outcome of antiviral treatment in this patient population [27, 28].

Controversial results are reported in the literature regarding text message interventions in patients with HIV infection receiving antiretroviral treatment in whom, similar to HCV patients receiving DAAs, daily treatment intake is crucial for treatment efficacy: while some studies concluded that regular text message reminders improve compliance to treatment and clinical visits [29, 30], a more recent review reported that the influence of daily text message reminders on treatment intake was inconclusive, but interactive message interventions led to increased adherence to treatment [31]. In our patient cohort, any contact with the HCV-Phone hotline improved treatment initiation, but adherence to clinical visits was similar to those of patients that were not in contact with the HCV-Phone. As a substantial part of patients in the HCV-Phone-related group showed poor adherence to follow-up visits in earlier treatment, adherence nonetheless may have improved through text or voice message reminders.

Our study has several limitations: firstly, some of the data analyzed in this study were collected retrospectively and therefore relied on the accuracy of the medical records. Secondly, due to the small sample size of patients with non-HCV-Phone-associated DAA treatments, comparison between both groups must be interpreted with caution. Thirdly, in our analysis we could not evaluate the intrinsic motivation for adherence to DAA therapy in a systematic way, precluding a fair comparison between the HCV-Phone-related group vs. HCV-Phone-unrelated treatment group. Finally, we were also unable to analyze the impact of contact frequency on adherence.

We conclude that the implementation of a physician-operated HCV-Phone hotline for patients with HCV and referring physicians is a low cost and low effort tool that reduces the barriers to HCV treatment evaluation, facilitates access to DAA therapy and possibly improves treatment efficacy in HCV patients with poor adherence.

## Abbreviations

DAA: Direct acting antivirals
ETR: End of treatment response
GT: Genotype
HCV: Hepatitis C virus
HIV: Human immunodeficiency virus
IDU: Intravenous drug abuse
IFN: Interferon
IQR: Interquartile range
ITT: Intention-to-treat analysis
MSM: Men who have sex with men
PrEP: Pre-exposure prophylaxis
PP: Per-protocol analysis
PWID: People who inject drugs
SVR: Sustained virological response
WHO: World Health Organization

## References

[1] Blach S, Zeuzem S, Manns M (2017). Global prevalence and genotype distribution of hepatitis C virus infection in 2015: a modelling study. Lancet Gastroenterol Hepatol.

[2] Degenhardt L, Peacock A, Colledge S (2017). Global prevalence of injecting drug use and sociodemographic characteristics and prevalence of HIV, HBV, and HCV in people who inject drugs: a multistage systematic review. Lancet Glob Health.

[3] Midgard H, Weir A, Palmateer N (2016). HCV epidemiology in high-risk groups and the risk of reinfection. j Hepatol.

[4] Organization. WHO (2017). Global Hepatitis Report 2017.

[5] Popping SB, Boucher C (2019). The global campaign to eliminate HBV and HCV infection: International Viral Hepatitis Elimination Meeting and core indicators for development towards the 2030 elimination goals. J Virus Erad.

[6] Kwo PY, Poordad F, Asatryan A (2017). Glecaprevir and pibrentasvir yield high response rates in patients with HCV genotype 1–6 without cirrhosis. j Hepatol.

[7] Mandorfer M, Schwabl P, Steiner S (2016). Interferon-free treatment with sofosbuvir/daclatasvir achieves sustained virologic response in 100 % of HIV/hepatitis C virus-coinfected patients with advanced liver disease. AIDS.

[8] Chromy D, Mandorfer M, Bucsics T (2019). High efficacy of interferon-free therapy for acute hepatitis C in HIV-positive patients. United European Gastroenterol J.

[9] Marshall AD, Pawlotsky J-M, Lazarus JV, Aghemo A, Dore GJ, Grebely J (2018). The removal of DAA restrictions in Europe—one step closer to eliminating HCV as a major public health threat. J Hepatol.

[10] Mandorfer M, Payer BA, Scheiner B (2014). Health-related quality of life and severity of fatigue in HIV/HCV co-infected patients before, during, and after antiviral therapy with pegylated interferon plus ribavirin. liver Int.

[11] Scheiner B, Schwabl P, Steiner S (2016). Interferon-free regimens improve health-related quality of life and fatigue in HIV/HCV-coinfected patients with advanced liver disease: a retrospective study. Medicine.

[12] Socias ME, Ti L, Wood E (2019). Disparities in uptake of direct-acting antiviral therapy for hepatitis C among people who inject drugs in a Canadian setting. liver Int.

[13] Schmidbauer C, Chromy D, Schmidbauer V (2020). Epidemiological trends in HCV transmission and prevalence in the Viennese HIV+ population. liver Int.

[14] Chromy D, Schmidt R, Mandorfer M, et al. HCV-RNA is commonly detectable in rectal and nasal fluids of patients with high viremia. Clin Infect Dis. 2020;71(5):1292–9. 10.1093/cid/ciz948. 10.1093/cid/ciz948 31562817

[15] Hoornenborg E, Achterbergh RCA, Schim van der Loeff MF (2017). MSM starting preexposure prophylaxis are at risk of hepatitis C virus infection. AIDS.

[16] Collins KM, Armenta RF, Cuevas-Mota J, Liu L, Strathdee SA, Garfein RS (2016). Factors associated with patterns of mobile technology use among persons who inject drugs. Subst Abus.

[17] Tofighi B, Leonard N, Greco P, Hadavand A, Acosta MC, Lee JD (2019). Technology use patterns among patients enrolled in inpatient detoxification treatment. J Addict Med.

[18] Coombes CE, Gregory ME (2019). The current and future use of telemedicine in infectious diseases practice. Curr Infect Dis Rep.

[19] Cao B, Gupta S, Wang J (2017). Social media interventions to promote HIV testing, linkage, adherence, and retention: systematic review and meta-analysis. J Med Internet Res.

[20] Plant A, Snow EG, Montoya JA, Young S, Javanbakht M, Klausner JD. Test4hepC: promoting hepatitis C testing to baby boomers using social media. Health Promot Pract. 2020;21(5):780–90. 10.1177/1524839919833987. 10.1177/1524839919833987 30854905

[21] Reiberger T, Ferlitsch A, Payer BA (2012). Noninvasive screening for liver fibrosis and portal hypertension by transient elastography—a large single center experience. Wien Klin Wochenschr.

[22] European Association for the Study of the Liver (2018). Electronic address eee and European Association for the Study of the L. EASL Recommendations on Treatment of Hepatitis C 2018. j Hepatol.

[23] Harris M, Rhodes T (2013). Hepatitis C treatment access and uptake for people who inject drugs: a review mapping the role of social factors. Harm Reduct J.

[24] Asselah T, Kowdley KV, Zadeikis N (2018). Efficacy of Glecaprevir/Pibrentasvir for 8 or 12 weeks in patients with hepatitis C virus genotype 2, 4, 5, or 6 infection without cirrhosis. Clin Gastroenterol Hepatol.

[25] Montes ML, Olveira A, Ahumada A (2017). Similar effectiveness of direct-acting antiviral against hepatitis C virus in patients with and without HIV infection. AIDS.

[26] Toyoda H, Atsukawa M, Watanabe T, et al. Real-world experience of 12-week direct-acting antiviral regimen of glecaprevir and pibrentasvir in patients with chronic hepatitis C virus infection. J Gastroenterol Hepatol. 2020;35(5):855–61. 10.1111/jgh.14874. 10.1111/jgh.14874 31609495

[27] Schmidbauer C, Schubert R, Schutz A (2020). Directly observed therapy for HCV with glecaprevir/pibrentasvir alongside opioid substitution in people who inject drugs-First real world data from Austria. plos One.

[28] Schutz A, Moser S, Schwanke C (2018). Directly observed therapy of chronic hepatitis C with ledipasvir/sofosbuvir in people who inject drugs at risk of nonadherence to direct-acting antivirals. J Viral Hepat.

[29] Finitsis DJ, Pellowski JA, Johnson BT (2014). Text message intervention designs to promote adherence to antiretroviral therapy (ART): a meta-analysis of randomized controlled trials. Plos One.

[30] Mayer JE, Fontelo P (2017). Meta-analysis on the effect of text message reminders for HIV-related compliance. aids Care.

[31] Shah R, Watson J, Free C (2019). A systematic review and meta-analysis in the effectiveness of mobile phone interventions used to improve adherence to antiretroviral therapy in HIV infection. BMC Public Health.

